# Supplementary material for: Reliable Fabrication of Graphene Nanostructure Based on e-Beam Irradiation of PMMA/Copper Composite Structure
Source: Materials (Basel). 2021 Aug 17;14(16):4634. doi: 10.3390/ma14164634 (PMC8401420; doi:10.3390/ma14164634)
Supplement: Supplementary file 1 [file materials-14-04634-s001.zip › materials-1330377-supplementary.pdf]

materials

MDPI

Article

Reliable Fabrication of Graphene Nanostructure Based on e-Beam Irradiation of PMMA/Copper Composite Structure

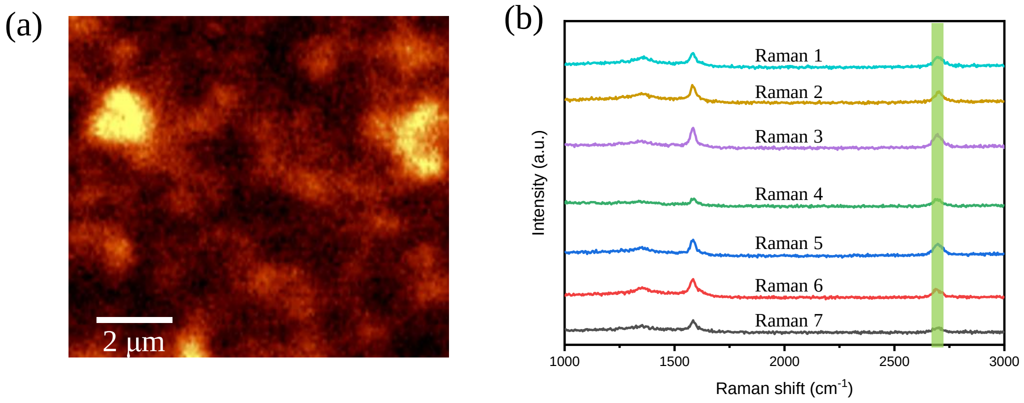

Figure S1. (**a**) Raman mapping for the area outline in Figure 3a at the 2D peak. (**b**) Raman spectra extracted randomly from Raman mapping area.

Just as shown in Figure S1, it is the Raman mapping at 2D peak for the area outline in Figure 3a. There are some differences at different positions maybe result from the irregular change of the number of layers and defects of graphene because of copper particles agglomeration. In order to explain the mechanism of the phenomenon, the Raman spectra was extracted randomly from Raman mapping area for further discussion. The graphene parameters, including 2D width, D/G intensity ratio and the 2D/G intensity ratio, was calculated in Table S1.

Table S1. A statistical evaluation of the graphene parameters from Figure S1b.

	2D width	I_D/I_G	I_{2D}/I_G
**1**	43.1	0.5	0.8
**2**	48.4	0.2	0.8
**3**	53.9	0.1	0.7
**4**	37.8	0.4	1.4
**5**	48.4	0.3	0.8
**6**	48.6	0.5	0.7
**7**	37.7	0.2	0.5

The 2D width of test areas in table 1 were varying from 37.7 cm^{-1} to 53.9 cm^{-1}, which means a multilayer graphene sheet on copper surface. Commonly, the I_D/I_G ratio and I_{2D}/I_G ratio depends on the number of layers. I_D/I_G ratio (0.1~0.5) and I_{2D}/I_G ratio (0.5~1.4) also indicates that multilayer graphene sample was acquired on copper surface. Besides that, the I_D/I_G ratio of sample is smaller than 1.0, which implies the defects and disordering is also lower than other substrates.
